# Reviews of Dental Literature

**Published:** 1905-11

**Authors:** 


### Reviews of Dental Literature.

Contribution to the Pathology and Treatment of Tic Douloureux of the Face.[1] By J. Besson (de Grenoble).

I report five observations of tic douloureux of the face which I have made during two years. Fortunately the patients have been willing to submit to a surgical treatment not often resorted to in cases of chronic neuralgia.

This treatment, however, is not new. In 1894 Dr. Jarre spoke in the *Revue de Stomatologie* of the pathology and treatment of tic douloureux, based on the latest works of stomatologists.

According to him it is not logical to attribute tic douloureux always to a central nervous origin. The frequent infection of the mouth, the composition and structure of the teeth, easily penetrated by the products of fermentation, the sensitiveness of these same teeth, the frequent lesions of the gums, of the alveolus, and the adjacent osseous tissues, appeared to explain sufficiently the nervous lesions of the adjacent tissues. It was here that we had to look for a pathology and the logical treatment of tic douloureux, which was probably caused by peripheral nerve-filaments of the trigeminus. In order to support his hypothesis, Dr. Jarre reports a series of tics cured through the extraction of all the teeth on the affected side, and especially the removal of the diseased alveolar walls.

The communication of Cruet at the International Congress of Medicine, 1903, and the theses of Gaumerais and Gillet confirmed this theory.

In December, 1902, Dr. Hermite de Grenoble sent a patient to me who suffered for thirteen years of an intermittent tic douloureux. The attacks recur, from month to month, lasting several days, sometimes weeks. When I saw him he was suffering from a severe paroxysm of pain. The right side of the face was affected by spasms, especially at the level of the eyelid and the lips. The lips were paralyzed, the saliva was constantly running from the

---

[1] Translated for this journal from the February Number, 1905, of the Revue de Stomatologie, by Clara M. Lötschert, D.D.S.

mouth. The equally paralyzed tongue articulated with difficulty some vague sounds. The skin presented an unusual hyperæsthesia. The slightest contact of the right side of the face with a foreign body increased the pain. Here was, indeed, a typical case of tic douloureux of the face.

As predisposing cause, hereditary and personal, the history revealed only articular rheumatism.

I examined the mouth. It showed the characteristics of gouty condition,—congested and spongy gums inclosing some loose elongated teeth covered with tartar. Four teeth only remained in the superior maxilla, which was the seat of the neuralgia. I decided upon extraction, which was followed by the free use of hydrogen peroxide.

The conditions, however, remained unchanged; other remedies had to be resorted to. The idea that the bone was involved had presented itself from the very first. In order to generalize, we decided to direct our operation only to the superior maxilla, which seemed to be the seat of the trouble, especially the canine region. The slightest touch on the skin in this portion of the face brought on a severe attack of pain.

We applied the chisel and the bone forceps to the whole bony area situated between the sinus, the nasal fossa, and the supra-orbital canal. The operation was then completed with the removal of the alveolar walls behind the canine region.

One incident during the operation caused some annoyance,—the accidental opening of the sinus.

The result was immediate. In waking, the patient declared himself entirely relieved; the spasms and the pain had disappeared. Our operative interference had occurred during one of the paroxysmal crises.

The cure continues until this very moment. But an infection of the sinus through the buccal opening necessitated frequent antiseptic injections. Dr. Hermite was called to close the opening by a suture; a cutaneous entrance was established for our preventive measures, the protection of the mouth from bacterial influence.

The patient, the janitor of the faculty, sleeps and eats normally. During the past two years he has hardly felt a vague shooting sensation in the supra-orbital region. His awaking in the morning is now a pleasant sensation after the horrible nightmare of thirteen years.

This clinical observation called our attention to two points: first, the fixed point of the pain located on a level with the canine fossa; second, a peculiar circumstance revealing itself during the operation,—the compact, ivory-like appearance, the excessive hardness of the osseous tissue in the canine region. Both are of special value when we consider the result obtained through the operation. New cases were studied more methodically.

Two months later, in January, 1903, M. C., sent by Dr. Perriol de Grenoble, presented himself.

The same symptoms—paroxysms of pain dating back fifteen years with rare intervals; the whole organism considerably weakened by intolerable suffering.

The mouth was intact in spite of the advanced age of sixty-five. Not one tooth was missing, but it showed the characteristics of a long-standing pyorrhœa alveolaris.

Three to four teeth were suspected and extracted without modification of the pain. Definitive interference followed a few days later,—the extraction of all the teeth on the affected side and the "resection" of the osseous part of the canine region with the help of Dr. Perriol.

The result was marvellous. Previous to this operation the inferior maxillary nerve had already been removed by Professor Jaboulay de Lyon without marked effect. Two years later Dr. Perriol removed the supra-orbital nerve. After each of these operations the patient, in waking from the anæsthesia, experienced the sensation that the intensity of the pain was the same as before. After our surgical interference, however, he awoke with the conviction that he was cured, for all pain had disappeared.

Unfortunately, the weakened constitution of the patient could not stand the violent shocks of the treatment. He died a month later from phlebitis of the lower limbs.

Here again had been the symptoms of the tic douloureux,—spasms, pain located in the canine region. All had yielded to the surgical operation performed during a period of the paroxysms. The maxilla showed at the mentioned area an excessive compactness. The mouth had been long since the seat of pyorrhœa alveolaris.

In May of the same year, with the assistance of the doctoress Bruyant and that of my brother, I performed the same operation on a woman of the suburbs, fifty-five years of age, suffering for two years from facial neuralgia of light spasmodic character. Extracted eleven teeth on the affected side. Then a month later, the pain having continued meanwhile, removed the osseous part of the canine region. Immediate cessation of the pain without recurrence. The same observation as before regarding the location of the pain and the compactness of the bone.

In August, operation on a woman sixty years old. Mouth very much affected by chronic pyorrhœa; neuralgia dating two years.

Three or four remaining teeth were extracted; a few days later, resection of canine area. The result was even more complete. We have seen our patient again. She does no longer suffer.

The last operation on record took place August, 1903. At the distance of one month we performed two partial resections of the superior maxilla on a woman fifty years of age, suffering for ten years from tic douloureux. The first surgical interference brought no relief. At the second we approached the supra-orbital nerve. She felt a slight decrease of pain for a period of three months. But the pain has recurred since then. Grateful for the relief, she came back desiring that we should go farther. Here again we had to do with a mouth of arthritic character undermined by pyorrhœa alveolaris. Once more the paroxysms appeared to take their origin on a level with the canine fossa, and the superior maxilla was at that period very compact. It remains to state that in three cases of those operated upon the teeth showed signs of old arthritis with calcified pulps.

From all these operations I have only marked out the most striking points. I will repeat them in a few words.

1. The neuralgia or chronic facial "névritis" is an affection of advanced age. It seems to manifest itself more in gouty persons whose tissues undergo senile or sclerotic changes.

2. The seat of the pain seems to be more that portion of the trigeminus which expands on a level with the canine fossa, that is to say the anterior dental branch.

3. The "névritis" of these nerve extremities is accompanied by a marked compactness of the bone tissues.

4. In five cases operated upon the canine fossa, the results have obviously been far superior to those obtained by extirpation of the nerves.

5. We conclude with Dr. Jarre that the chronic facial névritis appears to be the consequence of oral infection in gouty persons and of the osseous transformations which take place in the length of time. It is a neuralgia through infection and pressure of bone, comparable to the dental pulpitis.

6. We agree, with Jarre, Cruet, Gaumerais, Gillet, when they regard tic douloureux in the majority of cases as a peripheral "névritis." We consider a surgical operation upon the peripheral region logical; one, limited to the alveolar region, insufficient.

7. We add that it must be made on the superior maxilla in general; in particular, on the canine fossa. Our clinical observations prevent this, while the following anatomical facts reinforce our opinion.

If we consider the character of the superior and inferior dental plexus, we find that throughout the nerve-endings detach themselves from the trunk in order to disappear almost immediately in the dental canal, except on one point. The anterior and the middle dental branches, branches of the superior maxillary nerve take their origin before the entrance of the maxillary nerve in the superior orbital sinus. From this they take their course through two special bony canals enclosed in the thickness of the wall of the sinus. Thus we have here two long osseous canals, each embracing a nerve branch, being at the same time in direct connection with the alveoli of the incisors and canines, also with the sinus.

When one knows the frequency of chronic alveolar infections in arthritic patients, when one thinks of the frequent existence of obscure (ignored) sinuses, then it is permitted to think that the osseous tissue transforms itself under these influences; that it slowly compresses (smothers) the nerve branches of which the pulp extremities are often already dead.

At any rate, this hypothesis presents itself after having seen the dense, compact, ivory-like aspect of these tissues, the calcification of the pulp, and, above all, the cessation of the pain after the operation.

For us tic douloureux of the face is a peripheral nervous affection; it is curable through a surgical operation upon the canine region.

Why use Low-Carat Solder on New Dental Work? By William H. Trueman, D.D.S., Philadelphia.

The time was, and not so very long ago, when gold solder kept for sale at the dental depots was so brittle and so broken that it might be handled with a scoop. This is now changed. Firms catering to the wants of dental mechanics have brought skill and science to bear upon their metallurgical departments, and as a result the needed precious metals are obtainable in better form and in greater variety. The tendency, however, to debase the solders, while no doubt in response to a demand for solder of easy flowing qualities, is not to be commended. The fault, however, is not with them, but is due to faulty teaching in our schools and text-books. The time was when solders of a standard carat were for sale; now they are advertised and branded, not as of a certain carat, but as *solder for a certain carat plate,* special stress being laid upon their easy flowing qualities and good color, rather than upon their fineness. The reduced price plainly indicates that they are debased.

In an excellent text-book upon prosthetic dentistry, now before me, I find the following formula for a gold solder, which, it states, "may be used in connection with eighteen or twenty carat plate:"

6 dwts. pure gold.
2 dwts. rosette copper.
1 dwt. fine silver.

That such a formula should be published in a dental text-book of so recent a date (1897) is a disgrace, and yet it is a fair sample of those set before dental students by their class teachers and their text-books in this year of grace, 1905, copied without change from ancient text-books.

In the first place, it is not explicit, nor yet in accord with usage in scientific works. Why should the reader be compelled to work out a mathematical problem in order to obtain information that should be conveyed by the formula itself?

Let it read:

16 parts (or dwts. or grains, it is immaterial which) pure gold;

$5\frac{1}{3}$ parts rosette copper;

$2\frac{2}{3}$ parts fine silver; and the reader knows at once that the resulting solder is sixteen carats fine. It may be used, of course, upon eighteen- or twenty-carat plate; but why should such low-carat solder ever be used in connection with eighteen- or twenty-carat plate? This is followed by two others, one a fraction over fifteen carats fine, and another eighteen, both naming gold coin and brass as components. Now let me ask, What is "rosette copper"? What is "gold coin"? What is "brass"? When this formula was first written, some seventy or more years ago, "rosette copper" might possibly have been some special brand of that metal; it has no meaning now. Brass, fine brass, brass pins, etc., are of as uncertain composition as is a low grade of boarding-house hash. Regarding "gold coin," nothing is certain beyond its containing a definite amount of pure gold. No doubt, when the mouth blow-pipe was in general use, with the well-remembered alcohol lamp, that now and again blew up, or more frequently "went out," like those of the unwise virgins, and from the same cause, before the soldering operation was completed, or in prolonged operations blinded one by its irritating fumes, a solder fusing at a few degrees less heat was appreciated. All that is now of the past, and it is high time that expedients made necessary by meagre facilities were erased from dental college teaching and dental text-books.

All the needed metals—gold, silver, copper, and zinc—are now readily attainable, as nearly absolutely pure as chemical processes carefully conducted can make them; and they only should be used. The difference in cost between pure gold and pure silver, and coin gold and coin silver, is trifling. Pure gold costs, at the refiners, one dollar and ten cents per dwt. for any amount less than an ounce, and twenty-one dollars an ounce. This never changes. Pure silver fluctuates; its present price is about seventy-five cents an ounce; pure copper is about twenty cents an ounce; pure zinc about fifteen cents an ounce. The base metals may be obtained from dealers in fine chemicals, who provide these metals for use in delicate chemical processes. There is no excuse for using, and no excuse for advising the use of commercial metals or alloys in making either plate or solder for dental use. They all contain harmful impurities.

Now, it is not every dentist who has facilities for making his own plate or solder, and when it can be purchased from reliable parties it is better to do so. It is well, however, to understand that eighteen-carat gold solder and solder for eighteen-carat gold plate are not the same. The latter is usually about two points lower carat.

If in the formulas for plate and solder given in the text-books the number of grains of the precious metal in each pennyweight is first given, the formula itself announces the carat. It is rather more scientific to state the parts in thousands; either, however, is explicit. The use of alloys should be discarded. There is no advantage in their use; they are confusing and unreliable.

The following formulas I use in my own laboratory. They are all I need for new work, and I find them quite satisfactory. They flow freely, have good color, and are as tough and pliable as plate. Between their fusing point and that of the metals for which they are intended there is ample margin. The eighteen-carat will flow freely upon silver plate, and the twenty-two-carat upon eighteen-carat plate without in either case the plate being overheated. This is my test of their fusibility.

Twenty-two-carat gold solder:

22 parts pure gold;

1 part pure copper;

1¼ parts pure zinc.

Eighteen-carat gold solder:

18 parts pure gold;
3 parts pure silver;
2 parts pure copper;
1½ parts pure zinc.

Silver solder:

19 parts pure silver;
1 part pure copper;
4 parts pure zinc.

There is a slight loss of zinc, especially in making gold solder, not from oxidation, as the books generally state, but from volatilization, which is quite a different thing.

A fraction of a grain is added to allow for this. If pure metals are used, there is so little oxidation that as a rule the crucible from which the solder is poured is left perfectly clean; so clean, indeed, that they are promptly broken to avoid the temptation of using them again; a dangerous experiment, as the borax usually penetrates the bottom and causes them to leak when used a second time.

Either in safety to the porcelain teeth or to the plate, the slight difference in the fusing point between the high and low grade solder is of no practical advantage, and for new work a solder of a lower carat than the plate is inexcusable. The lower-grade solders are occasionally required in making repairs, and should be used for that purpose only.

Silver, being more fusible, requires a solder a little less fine, but there is no excuse for using a silver solder composed of equal parts of silver plate and brass.

While zinc is a base metal, and is credited with causing brittleness when alloyed with the precious metals, the character it has acquired as a component of precious metal alloys is not deserved. It is due to the impurities found associated with it rather than to the metal itself.

Notwithstanding that the solder sold as solder for eighteen-carat plate is but little if any finer than sixteen-carat, I am told by dealers that many dentists when purchasing eighteen-carat gold purchase sixteen-carat solder to use with it, so that as a matter of fact they are using on new work fourteen-carat solder. I wonder why! It cannot be economy; the difference in cost is too insignificant. I take it to be ignorance, or it may be that they do not possess proper heating arrangements, or lack effective blow-pipes.

With the advent of crown- and bridge-work, a handy form of blow-pipe has come into general use. For small, delicate operations they are very convenient, especially when the work is done in the office on an office work-bench. None that I have tested have proved satisfactory for serious work. The old-fashioned large gas flame and the old-fashioned blow-pipe, operated with a foot-pump or bellows, may make things hot on a warm day; may occasionally singe the hair or burn the fingers; but when their use is once mastered, soldering with honest, high-grade solder is a pleasure.

As gold solders are now graded, a solder two carats higher than the plate should always be used. It would be better, however, if the dealers would cease to equivocate, and sell their gold solders for what they really are.

Another reform. Our text-book writers should discard all the old out-of-date formulas. There is no necessity for a multiplicity of formulas, one for each grade is amply sufficient; and they should teach that the plate and solder should be of the same fineness.

As before remarked, the difference in the fusing point of solder differing slightly in carat is no help to the workman constructing a new denture, but it makes a great deal of trouble at times, when that piece of work requires repairs or alterations, and these are attempted with "honest" solder.—*Dental Office and Laboratory.*

